# Investigating marine bio‐calcification mechanisms in a changing ocean with in vivo and high‐resolution ex vivo Raman spectroscopy

**DOI:** 10.1111/gcb.14579

**Published:** 2019-02-20

**Authors:** Thomas M. DeCarlo, Steeve Comeau, Christopher E. Cornwall, Laura Gajdzik, Paul Guagliardo, Aleksey Sadekov, Emma C. Thillainath, Julie Trotter, Malcolm T. McCulloch

**Affiliations:** ^1^ Oceans Graduate School The University of Western Australia Crawley Western Australia Australia; ^2^ Oceans Institute at The University of Western Australia Crawley Western Australia Australia; ^3^ ARC Centre of Excellence for Coral Reef Studies Crawley Western Australia Australia; ^4^ School of Molecular and Life Sciences, TrEnD Laboratory Curtin University Bentley Western Australia Australia; ^5^ Centre for Microscopy, Characterisation and Analysis The University of Western Australia Crawley Western Australia Australia; ^6^ School of Biological Sciences The University of Western Australia Crawley Western Australia Australia; ^7^ School of Earth Sciences The University of Western Australia Crawley Western Australia Australia; ^8^ Present address: Sorbonne Université, CNRS‐INSU, Laboratoire d'Océanographie de 30 Villefranche 181 chemin du Lazaret, F–06230 Villefranche‐sur‐mer France; ^9^ Present address: School of Biological Sciences Victoria University of Wellington Wellington New‐Zealand

**Keywords:** calcification, coralline algae, corals, foraminifera, in vivo, ocean acidification, otoliths, Raman spectroscopy

## Abstract

Ocean acidification poses a serious threat to marine calcifying organisms, yet experimental and field studies have found highly diverse responses among species and environments. Our understanding of the underlying drivers of differential responses to ocean acidification is currently limited by difficulties in directly observing and quantifying the mechanisms of bio‐calcification. Here, we present Raman spectroscopy techniques for characterizing the skeletal mineralogy and calcifying fluid chemistry of marine calcifying organisms such as corals, coralline algae, foraminifera, and fish (carbonate otoliths). First, our in vivo Raman technique is the ideal tool for investigating non‐classical mineralization pathways. This includes calcification by amorphous particle attachment, which has recently been controversially suggested as a mechanism by which corals resist the negative effects of ocean acidification. Second, high‐resolution ex vivo Raman mapping reveals complex banding structures in the mineralogy of marine calcifiers, and provides a tool to quantify calcification responses to environmental variability on various timescales from days to years. We describe the new insights into marine bio‐calcification that our techniques have already uncovered, and we consider the wide range of questions regarding calcifier responses to global change that can now be proposed and addressed with these new Raman spectroscopy tools.

## Graphical abstract

Newly developed in vivo and high‐resolution Raman spectroscopy analyses of marine calcifying organisms enable characterization of their mineralogy and calcification mechanisms. Insights from these new Raman spectroscopy tools are helping us understand the sensitivity of these important organisms to ocean warming and acidification.

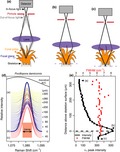

## 1 INTRODUCTION

The production of calcium carbonate (CaCO_3_) by marine calcifying organisms plays a key role in the global carbon cycle and influences the chemistry of the ocean (Berelson et al., 2007). Biological calcification (or “bio‐calcification”) is performed by a variety of species in different environments that is manifested, for example, as the immense geologic reef structures built by corals and coralline algae, as well as being essential to calcareous plankton such as foraminifera and to the formation of calcium carbonate structures in the inner ears of fishes. The evolutionary history of bio‐calcification extends back hundreds of millions of years and is found in multiple kingdoms, reflecting the many benefits that calcium carbonate mineralization provides, including skeletons for protection, structural support, and balance (Knoll, 2003). This widespread utility of calcification by marine organisms has depended on the favorability of calcium carbonate to form in seawater, with saturation states (Ω) of the calcium carbonate minerals aragonite and calcite exceeding one (i.e., oversaturation) in surface waters for hundreds of millions of years (Tyrrell & Zeebe, 2004; Zeebe, Ridgwell, & Zachos, 2016).

However, with humans now emitting CO_2_ at rates that are likely unprecedented even on geologic timescales (Zeebe et al., 2016), the carbonate chemistry of the surface oceans is fundamentally changing in ways that many calcifying taxa have not experienced in their evolutionary history (Hönisch et al., 2012; Tyrrell & Zeebe, 2004). Some previous mass extinctions of marine calcifying species have occurred under changes in ocean carbonate chemistry (especially with respect to Ω) that may have been less than the changes expected within the coming centuries due to the rapid release of CO_2_ from human activities (Hönisch et al., 2012; Ridgwell & Schmidt, 2010; Veron, 2008). Therefore, although the extant taxa may respond differently from those affected during previous mass extinctions, modern CO_2_‐driven ocean acidification raises serious concerns about the future survival of calcifying species as well as the persistence of marine bio‐calcification in general.

Forecasting the impacts of ocean acidification on marine calcifying organisms requires an understanding of the mechanisms by which they produce their calcium carbonate structures. It is clear that a range of calcification mechanisms exist because various organisms build their shells and skeletons with different calcium carbonate minerals. For example, aragonite is utilized by many corals and fishes, whereas calcite is utilized by most coralline algae and foraminifera. However, the classic notion that such organisms directly precipitate these different minerals has come under question. Recent studies have speculated that corals build their aragonitic skeletons from amorphous calcium carbonate (ACC) particles (Von Euw et al., 2017; Mass et al., 2017), that planktonic foraminifera form vaterite as a precursor to calcite (Jacob, Wirth, Agbaje, Branson, & Eggins, 2017), and that some coralline algae stabilize their skeletons with dolomite and magnesite (Nash et al., 2011, 2013). These unexpected findings imply greater complexities in marine bio‐calcification and underscore the need for further investigations into the mechanisms by which calcification occurs. Additionally, the response of various calcifiers to ocean acidification likely depends on their mineralogy due to the different solubilities of carbonate minerals (Morse, Mucci, & Millero, 1980).

We present Raman spectroscopy techniques for investigating the mineralogy of calcium carbonate structures in marine calcifying organisms with two complementary approaches: in vivo and high‐resolution ex vivo mapping. Raman spectroscopy exploits the inelastic scattering of light to characterize sample mineralogy and the chemical bonding environment of crystals forming the shells and skeletons (Smith & Dent, 2005). Since Raman spectroscopy is highly sensitive to the various calcium carbonate minerals (Dandeu et al., 2006; Perrin et al., 2016; Stolarski et al., 2016), it is an ideal tool for investigating precursor phases within calcified structures. Furthermore, Raman spectroscopy has recently been applied to aragonitic corals to quantify the calcifying fluid aragonite saturation state (Ω*_Ar_*) and its sensitivity to ocean acidification (DeCarlo et al., 2017). A key advantage of our in vivo approach presented here is the non‐invasive nature of the technique, which removes the need for sample preparation or sacrificing the organism, two potential issues which could have confounded some previous results. We apply in vivo Raman spectroscopy to two species of aragonitic corals, a foraminifer, and a coralline alga. Additionally, we create high‐resolution (micron‐scale) ex vivo Raman maps of the calcium carbonate structures of a tropical coral, a deep‐sea coral, a fish otolith, a foraminifer, and a coralline alga.

## 2 MATERIALS AND METHODS

### 2.1 Sample collections

#### 2.1.1 Specimens for in vivo Raman spectroscopy

Living *Pocillopora damicornis* and *Acropora yongei* corals, and a benthic foraminifer (*Amphisorus *sp.) were collected from Rottnest Island in Western Australia (32.02°S, 115.52°E; see Ross, Falter, Schoepf, and McCulloch (2015) for a detailed map of the study area). Two different *A. yongei* colonies were used (hereafter referred to as *A. yongei *1 and 2). Additionally, living coralline algae (*Hydrolithon reinboldii*) were collected from Tallon Island in the Kimberley region of Western Australia (16.41°S, 123.12°E; see Cornwall et al. (2018) for collection details). All specimens were maintained in plastic or glass aquaria at the Waterman's Bay marine research facility of the University of Western Australia. Branch tips (1–2 cm) of the two coral species were broken from each colony, whereas the entire living foraminifer was used for in vivo analyses. The *Hydrolithon reinboldii* specimens produced offspring that recruited onto the sides of plastic aquaria, and a piece of an aquarium containing a living recruit was removed for subsequent analysis.

#### 2.1.2 Specimens for high‐resolution ex vivo Raman spectroscopy mapping

A living colony of *Stylophora pistillata* was collected at 2 m depth from Ningaloo Reef near Coral Bay, Western Australia, the tissue removed with a water jet, and the skeleton dried in an oven at 50°C for 24 hr. A branch tip was broken from the skeleton and a petrographic thin section was prepared.

A deep‐sea cup coral, *Desmophyllum dianthus*, was collected from a depth of 675 m within the Antarctic Intermediate Waters of the Perth Canyon offshore Western Australia, during the oceanographic cruise FK20150301 (31.55°S, 115.05°E; see Trotter et al., 2018). The coral was soaked in purified (Milli‐Q) water for several hours to remove organic tissue, and the skeleton was then dried overnight in an oven at 40°C. A septum and the adjacent wall were removed using a dental drill and diamond disk, set in epoxy resin, polished to expose the calcification centers, and sonicated in Milli‐Q water and then AR grade methanol.

The coralline alga *Sporolithon durum* was collected from Bremer Bay (34.4°S, 119.4°E; see Ross, Schoepf, DeCarlo, and McCulloch (2018) for a description of the collection site).

A juvenile planktonic foraminifer,* Orbulina universa*, was collected off the coast of New South Wales, Australia and cultured in the laboratory at Australian National University during 2007 following the protocol described in Hori et al. (2018). The specimen formed a spherical chamber in the aquarium and underwent gametogenesis 8 days later. The shell of the *O. universa* specimen was cleaned in deionized water and embedded in resin for Raman spectroscopy.

Finally, we used a sagittal otolith from a coral reef fish (*Pseudochromis fuscus*) caught on Ningaloo Reef. Annual bands in the otolith were used in a previous study to identify the specimen age (Thillainath, McIlwain, Wilson, & Depczynski, 2016). The sample was previously cut and ground with a flat surface glued to a glass slide. We conducted our Raman measurements through the glass slide to image the flat face cut through the center of the otolith.

### 2.2 In vivo Raman spectroscopy

Raman spectroscopy measurements were made in vivo on coral (*P. damicornis* and *A. yongei*), a coralline alga (*H. reinboldii*), and a benthic foraminifer (*Amphisorus *sp.). Each specimen was placed in seawater (sourced from Waterman's Bay, Western Australia), either in a petri dish (foraminifer), or in a cup with a water‐circulating pump that was turned off several minutes prior to Raman measurements. Generally, Raman spectra were collected in the dark, although measurements in the light are also possible (see Figure S1). Measurements were made with a WITec Alpha300 RA+ confocal Raman microscope system with a 785 nm laser source, a 20X submersible objective with 0.5 aperture, a CCD detector maintained at −60°C, and either a 600 or 1,200 mm^−1^ grating. Different gratings were used to test if the method was generalizable because some applications may require the broader spectral coverage of a 600 mm^−1^ grating (e.g., to identify “lattice‐mode” peaks; Dandeu et al., 2006), despite its lower spectral resolution (discussed below). Repeated analyses of a silicon chip were conducted for wavenumber calibration, and reported wavenumbers have been calibrated to the primary silicon peak at 520.7 cm^−1^.

**Figure 1:**
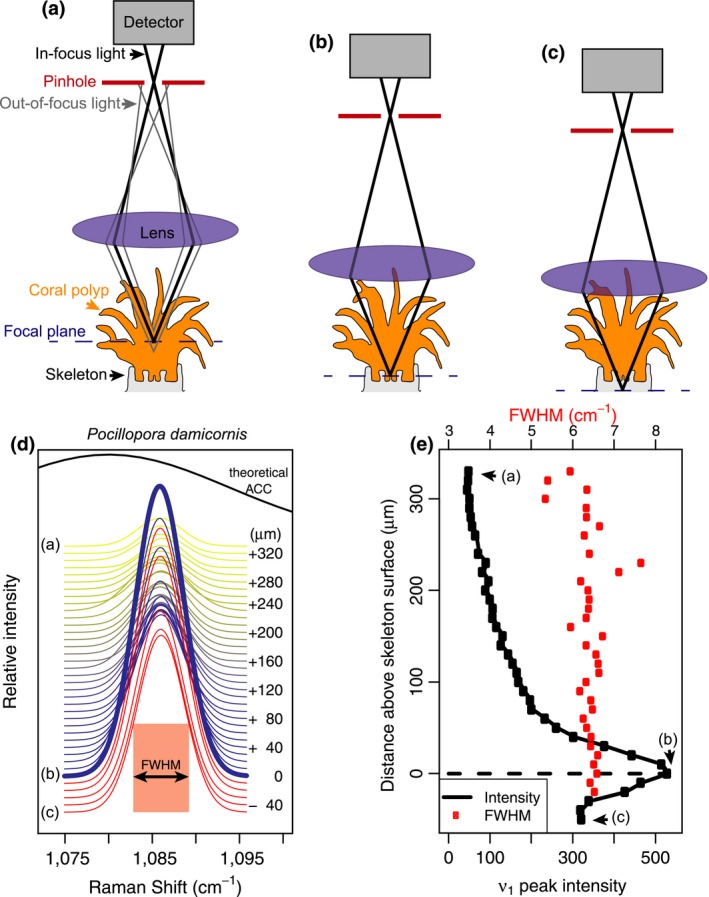
Schematic illustrating in vivo Raman profiling of living corals. (a) Initially, the Raman system is focused near the outer tissue layer. A pinhole (red) in the confocal Raman microscope blocks out‐of‐focus light (gray lines), allowing only in‐focus light (thick black lines) from a narrow focal plane (blue dashed line) to reach the detector. As the microscope objective is lowered, the focal plane reaches the skeleton (b) and then the focal plane extends slightly beneath the skeletal surface (c). (d) Raman ν_1_ peaks collected as the microscope focus moved from the outer tissue to the skeleton (yellow to blue), and out‐of‐focus beneath the skeletal surface (red). The thick blue line corresponds to the spectrum with the greatest ν_1_ peak height. The letters to the left of the peaks correspond to schematic panels (a–c) above, and the numbers to the right of the peaks indicate the distance of the focal plane above the skeleton surface. The black curve shows the expected appearance of an amorphous calcium carbonate (ACC) peak based on Wang et al. (2012). (e) Intensity of the ν_1_ peak (black) and its Full Width at Half Maximum (FWHM; red) for the spectra shown in (d). The detection of the ν_1_ peak and its FWHM through the coral polyp arises from weak signals of scattered and/or out‐of‐focus light, not from aragonite crystals within the tissue [Colour figure can be viewed at http://www.wileyonlinelibrary.com/]

Living specimens were placed in seawater and examined under the microscope. The objective was lowered into and then raised out of the seawater several times to remove air bubbles on the lens before final submersion into the seawater. Initially, the focal plane was set near the outer tissue layer of each specimen under bright‐field illumination. Next, the laser was turned on and Raman spectra were recorded every 3 to 5 s while incrementally moving the focus downwards into the tissue and toward the skeletons (Figure 1a–c). The intensity of the ν_1_ peak, which represents the symmetric stretching of C–O bonds in all calcium carbonate minerals, was used to identify the location of the skeleton surface. We assumed that the spectrum with maximum intensity of the ν_1_ peak corresponded to where the confocal plane was most closely aligned with the top of the skeleton (Figure 1d–e). For corals, we used the peak width (full width at half maximum intensity, or FWHM; see Figure 1) of the ν_1_ peak to determine Ω*_Ar_* of the calcifying fluid from which the skeleton recently formed, based on the previously described calibration between FWHM and Ω*_Ar_* of abiogenic aragonites (DeCarlo et al., 2017). This calibration is based on the finding that aragonite precipitating from higher supersaturation is more disordered (higher FWHM) due to either incorporation of impurities or lattice defects that occur under rapid crystallization. For the calcitic foraminifer and coralline alga, we used the ν_1_ peak wavenumber to estimate the skeleton %Mg (Borromeo et al., 2017; Perrin et al., 2016). Additionally, we calculated the residual FWHM between measured FWHM and that calculated based on a calibration between FWHM and %Mg (Perrin et al., 2016).

### 2.3 Evaluation of potential effects of seawater on carbonate ν_1_ FWHM

We tested whether immersing coral skeletons in seawater influenced the Raman ν_1_ peak. Specifically, we tested whether ν_1_ FWHM of a single sample was different when measured in air or seawater. We placed a *Porites* sp. coral skeleton in an empty cup and collected 10 Raman spectra with the 1,200 mm^−1^ grating and the submersible objective. Next, we filled the cup with seawater without moving the skeleton or adjusting the microscope focus, and collected an additional 10 spectra.

### 2.4 High‐resolution ex vivo Raman spectroscopy mapping

Samples for high‐resolution ex vivo mapping were analyzed with the same Raman system, except using a non‐submersible 20X objective with a 0.5 numerical aperture. All measurements were made with a 1,200 mm^−1^ grating. Spatial resolution ranged from 0.25 to 15 µm, and integration times from 0.3 to 2.5 s, depending on the sample (Table S1). As with the in vivo measurements, we used ν_1_ FWHM as a proxy for Ω*_Ar_* in corals, and for calcitic samples, we calculated the %Mg (from ν_1_ wavenumber), ν_1_ FWHM, and ν_1_ residual FWHM (after accounting for Mg).

### 2.5 NanoSIMS

We used nanoscale secondary ion mass spectrometry (NanoSIMS) to map the distribution of Mg/Ca in the *S. pistillata* coral and *O. universa* foraminifer. These were the two specimens mapped with Raman at sufficiently small scales to compare directly with NanoSIMS. The NanoSIMS maps serve to confirm the presence of micro‐banding features in these samples, and especially to validate the interpretation of Mg/Ca in the foraminifer. Details of the NanoSIMS measurements can be found in the Supporting Material.

## 3 RESULTS

### 3.1 Effects of seawater on carbonate ν_1_ FWHM

The carbonate ν_1_ FWHM of the *Porites* sp. skeleton measured in air was 3.214 ± 0.003 (1σ)cm^−1^, which translates to Ω*_Ar_* of 7.2 ± 0.1 (DeCarlo et al., 2017). In comparison, ν_1_ FWHM of the same *Porites* sp. skeleton measured in seawater was 3.24 ± 0.01 cm^−1^, or a Ω*_Ar_* of 7.6 ± 0.2. A two‐sample *t* test showed that the difference in FWHM was significant (*p* < 0.05), although the difference in derived Ω*_Ar_* was relatively small (0.4 Ω*_Ar_* units). The greater FWHM from the measurements conducted in seawater is potentially due to a slight increase in instrument noise due to the scattered light passing through the seawater medium, as instrument noise is known to increase FWHM (DeCarlo et al., 2017; Nasdala et al., 2001). It is also possible that the seawater may have refracted the light to a different point on the skeleton surface, even though the sample itself was not moved. Thus, while there is potential for slight artificial increases in FWHM when conducting Raman spectroscopy measurements in seawater, the effect is relatively small and would be negligible for comparison of samples measured underwater.

### 3.2 In vivo Raman spectroscopy of marine calcifying organisms

**Figure 2:**
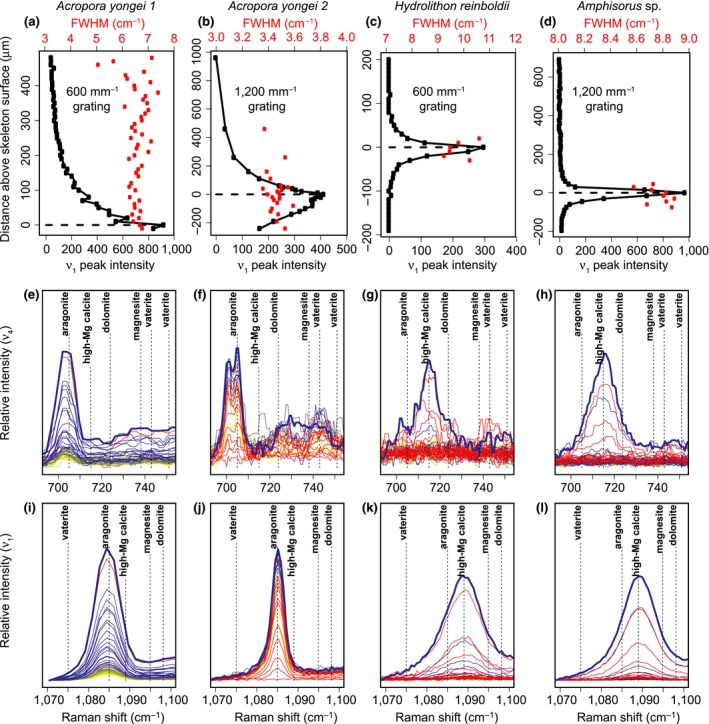
Application of in vivo Raman profiling to coral (*Acropora yongei*; a,b,e,f,i,j), a coralline alga (*Hydrolithon reinboldii*; c,g,k), and a foraminifer (*Amphisorus* sp.; d,h,l). (a–d) Profiles of ν_1_ peak intensity (black) and FWHM (red) (see Figure 1 schematic for additional details). The ν_1_ (e–h) and ν_4_ (i–l) peaks clearly reveal the two corals are aragonitic and both the coralline alga and foraminifer are calcitic. Colors in (e–l) follow the same scheme as in Figure 1d, with yellow to blue colors showing the transition from focus on the outer tissue to the skeleton, and red indicating out‐of‐focus below the skeletal surface. Peak positions for calcite (assuming 10% Mg), magnesite, and dolomite are based on Perrin et al. (2016) and the peaks for vaterite are based on Wehrmeister et al. (2009) and Melancon, Fryer, Gagnon, and Ludsin (2008) [Colour figure can be viewed at http://www.wileyonlinelibrary.com/]

Raman spectroscopy profiles conducted from the outer tissue layer downwards produced distinct maxima of ν_1_ peak intensity within hundreds of microns (Figures 1e and 2a–d). Analyses of the *P. damicornis* and the first *A. yongei* (“*A. yongei* 1”) coral specimens were conducted with a 600 mm^−1^ grating, which is less effective for quantifying Ω*_Ar_* due to the lower spectral resolution but captures a larger range of Raman shifts. Nevertheless, the ν_1_ FWHM of the spectra focused on the skeletal surfaces were 6.6 and 6.7 cm^−1^, respectively, which is consistent with Ω*_Ar_* < 20 (DeCarlo et al., 2017). The second *A. yongei* (“*A. yongei* 2”) specimen was analyzed with a 1,200 mm^−1^ grating, and its ν_1_ FWHM was 3.40 cm^−1^, equivalent to Ω*_Ar_* of 10 (DeCarlo et al., 2017). This is generally similar to that measured on *Acropora* skeletal samples (Comeau, Cornwall, DeCarlo, Krieger, & McCulloch, 2018; DeCarlo, Comeau, Cornwall, & McCulloch, 2018; DeCarlo et al., 2017), albeit on the lower end of the previously reported range. For the coralline alga *H. reinboldii*, the ν_1_ wavenumber was 1,088.63 cm^−1^, which corresponds to a Mg content of 10.8% (Perrin et al., 2016). The measured ν_1_ FWHM was 9.5 cm^−1^, compared to an expected FWHM of 8.2 cm^−1^ based on the Mg content (Perrin et al., 2016), leaving a “residual FWHM” of 1.3 cm^−1^. Consistent with this result, three previous studies of coralline algae skeletons calculated residual FWHM in the same way and reported values ranging from approximately 0.3 to 1.6 cm^−1^ (Comeau et al., 2018; Cornwall et al., 2018; McCoy & Kamenos, 2018). The *Amphisorus* sp. foraminifer had a ν_1_ wavenumber of 1,089.15 cm^−1^ (13.3% Mg), a measured FWHM of 8.83 cm^−1^, and a residual FWHM of −0.17 cm^−1^.

Inspection of the ν_1_ and ν_4_ peaks following previous studies (Dandeu et al., 2006; DeCarlo, Gaetani, Holcomb, & Cohen, 2015; Perrin et al., 2016; Wehrmeister, Soldati, Jacob, Häger, & Hofmeister, 2009) clearly revealed that all the corals are solely aragonitic, and both the coralline alga and the foraminifer are solely calcitic, with no other mineral phases detected in any of the profiles (Figure 2e–l). The ν_4_ peak of aragonite is a doublet centered at approximately 705 cm^−1^ (Dandeu et al., 2006), although the two peaks were distinguishable only with the 1,200 mm^−1^ grating (Figure 2f) but not the 600 mm^−1^ grating (Figure 2e). Regardless of the spectral resolution (600 mm^−1^ vs. 1,200 mm^−1^ gratings), the wavenumbers of the ν_1_ and ν_4_ peaks of all spectra in the coral profiles (Figure 2e,f,i,j) were consistent with aragonite. While the spectra from the coral profiles showed some increase in intensity between 720 cm^−1^ and 750 cm^−1^, the region where peaks for other carbonate minerals may be expected, there were no distinct peaks observed. Furthermore, there were no signs of any other ν_1_ peaks besides aragonite, suggesting that the slightly increased intensity between 720 cm^−1^ and 750 cm^−1^ is only an increase in the background, potentially due to fluorescence. The ν_1_ and ν_4_ peaks of the foraminifer and coralline alga profiles were clearly consistent with the sole presence of high‐Mg calcite. The profiles of all the organisms also showed no evidence of ACC. The absence of ACC is most clearly revealed by the fact that the ν_1_ FWHM are all less than 11 cm^−1^ (Figure 2a–d), which is unambiguously distinct from the FWHM of >20 cm^−1^ typical of ACC (Wang, Hamm, Bodnar, & Dove, 2012) (see also Figure 1d).

### 3.3 High‐resolution ex vivo Raman mapping of marine calcifying organisms

**Figure 3:**
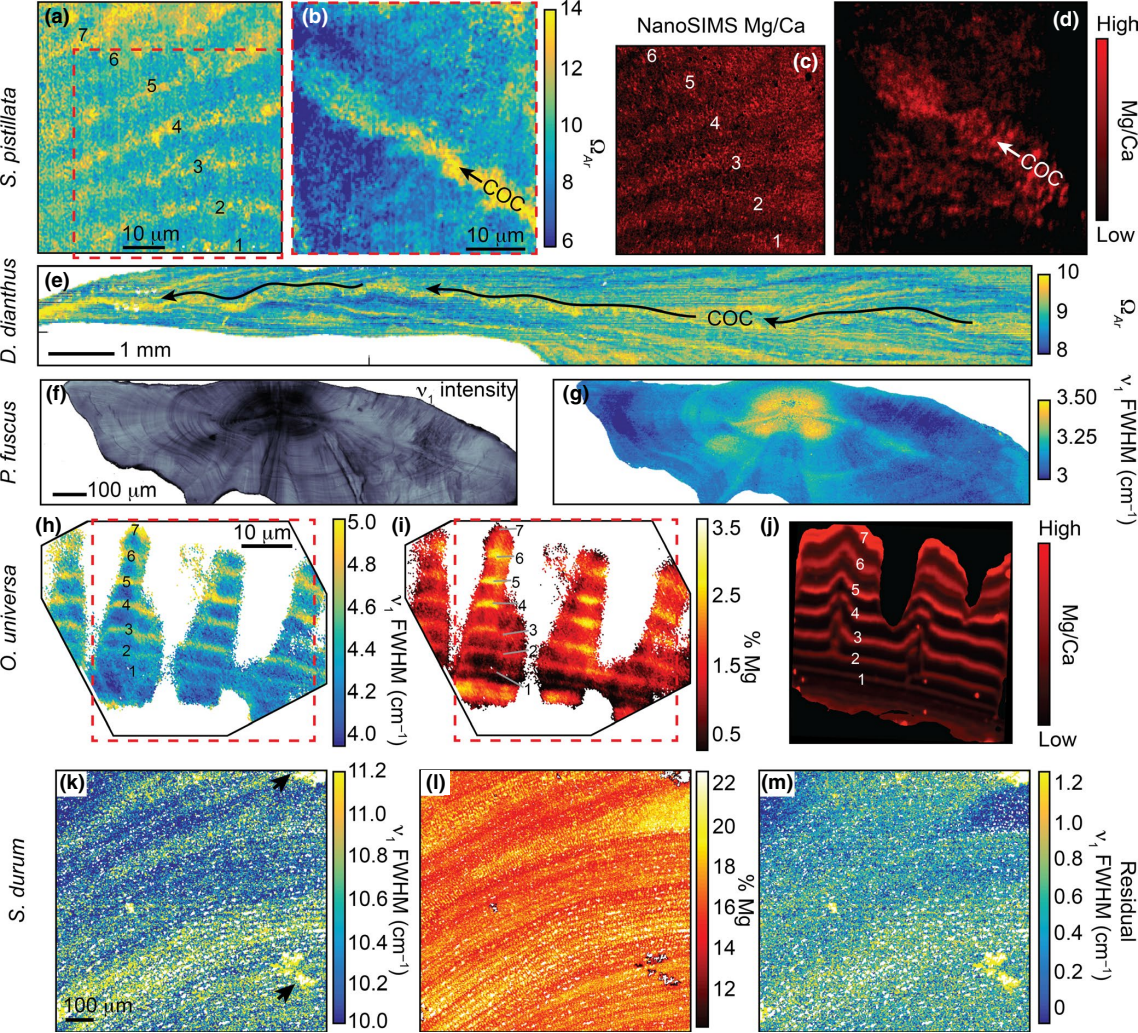
High‐resolution ex vivo Raman mapping of various marine calcifying organisms. (a–d) Shallow water coral (*S. pistillata*) with Ω_Ar_ mapped from ν_1_ FWHM (a,b), and Mg/Ca mapped with NanoSIMS (c,d; corresponding to a,b, respectively). (e) Deep‐sea coral (*D. dianthus*) Ω_Ar_. (f,g) Fish otolith (*P. fuscus*) maps of ν_1_ intensity (f) and ν_1_ FWHM (g). (h–j) Foraminifer (*O. universa*) ν_1_ FWHM (h), %Mg (i, from ν_1_ wavenumber), and relative Mg/Ca (j, from NanoSIMS). (k–m) Coralline alga *S. durum *ν_1_ FWHM (k), %Mg (l, from ν_1_ wavenumber), and ν_1_ residual FWHM (m, after accounting for the effect of Mg). Arrows in (k) indicate locations of minor aragonite presence. Red dashed boxes in a,b,h,i indicate the locations of NanoSIMS mapping. Numbers in the *Stylophora pistillata* and *Orbulina universa* maps aid in matching bands between images. “COC” indicates centers of calcification in the coral maps. Apparent differences in the shape of the foraminifer shell between Raman and NanoSIMS images are due to slight variations in the surface exposed to each measurement [Colour figure can be viewed at http://www.wileyonlinelibrary.com/]

Raman ex vivo mapping of corals, a fish otolith, a foraminifer, and a coralline alga revealed 10–100 μm banding patterns in each organism (Figure 3). For corals, micro‐banding patterns are reflected in the calcifying fluid Ω*_Ar_* (derived from ν_1_ FWHM). The ~10–20 μm wide oscillations of Ω*_Ar_* in the *S. pistillata* specimen (Figure 3b) may reflect daily bands. Additionally, we observed elevated Ω*_Ar_* at the centers of calcification (COCs; the yellow band indicating high Ω*_Ar_* in the center of the skeletal spine in Figure 3d), consistent with previous Raman analyses of *P. damicornis* (DeCarlo, Ren, & Farfan, 2018) and *Porites lutea* (Wall & Nehrke, 2012). The deep‐sea cup coral, *D. dianthus*, showed complex patterns of Ω*_Ar_*, with a central axis of centers of calcification in addition to fine‐scale Ω*_Ar_* banding throughout the skeleton (Figure 3e). While these banding patterns are of unknown temporal frequency, we note that deep‐sea corals grow their skeletons relatively slowly, with *Desmophyllum* annual extension rates ranging from 0.1 to 3 mm/year (Adkins, Henderson, Wang, O'Shea, & Mokadem, 2004; Cheng, Adkins, Edwards, & Boyle, 2000; Risk, Heikoop, Snow, & Beukens, 2002).

The fish otolith contained distinct banding patterns in ν_1_ peak intensity (Figure 3f). These bands may reflect both daily (near the nucleus) and annual (broader bands near the outer edge) growth accretions. Since the otolith is aragonite, ν_1_ FWHM could potentially be used to quantify Ω*_Ar_*; however, the published FWHM‐Ω*_Ar_* calibration was based on aragonite precipitating from seawater (DeCarlo et al., 2017). Corals likely precipitate aragonite from a seawater‐sourced solution (DeCarlo et al., 2017; Gagnon, Adkins, & Erez, 2012) with the elemental composition of their skeletons being similar to aragonite precipitated from seawater (DeCarlo et al., 2015; Gonneea, Cohen, DeCarlo, & Charette, 2017; Holcomb, Cohen, Gabitov, & Hutter, 2009). Conversely, otoliths mineralize from fluids tightly regulated by the fish, and their elemental composition is distinct from corals (Campana, 1999; Hathorne et al., 2013; Sturgeon et al., 2005; Yoshinaga, Nakama, Morita, & Edmonds, 2000). Therefore, we present the otolith ν_1_ FWHM in its measured units rather than deriving Ω*_Ar_* (Figure 3g). The variability of FWHM is dominated by a pattern of high FWHM in the nucleus and decreasing toward the outer edges, with some subtle banding superimposed onto this trend.

Raman analyses of calcitic specimens can also be used to estimate the molar %Mg by quantifying the wavenumber (i.e., position) of the ν_1_ peak (Perrin et al., 2016). In fact, the Mg content must be quantified before interpreting FWHM of calcite because the incorporation of varying amounts of Mg can impose changes in FWHM independent of other factors (Perrin et al., 2016). Our calcitic foraminifer (*O. universa*) and coralline alga (*S. durum*) exhibited clear banding patterns in ν_1_ wavenumber on spatial scales of order 10 μm (Figure 3h,k). Using an abiogenic calibration of ν_1_ wavenumber to %Mg (Perrin et al., 2016), these bands corresponded to Mg contents of approximately 0.5%–3% in the foraminifer and 15%–20% in the coralline alga. These estimates are generally consistent with previous measurements of Mg content in these species (Smith, Sutherland, Kregting, Farr, & Winter, 2012; Spero et al., 2015). Measured ν_1_ FWHM showed a nearly identical pattern to %Mg (Figure 3i,l), consistent with the notion that calcite ν_1_ FWHM variability can be dominated by the influence of Mg (see also Figure S2). Indeed, most of the ν_1_ FWHM banding disappeared when we calculated the residual FWHM (after removing the effect of Mg) (Figure 3m), yet some variations remain, such as a general decrease in residual FWHM from the bottom to the top of the map of the coralline alga. NanoSIMS Mg/Ca showed similar banding patterns as FWHM in the *S. pistillata* coral and in the *O. universa *foraminifer (Figure 3).

## 4 DISCUSSION

We present Raman spectroscopy techniques for investigating marine bio‐calcification mechanisms. With both in vivo and high‐resolution ex vivo mapping procedures, we show that Raman spectroscopy is a powerful tool for characterizing the mineralogy of a variety of marine calcifying organisms. Our initial applications demonstrate that Raman spectroscopy is suitable to address a range of questions at the forefront of current knowledge of marine bio‐calcification.

Raman spectroscopy has recently been applied to quantify the response of corals, coralline algae, and fish otoliths to simulated ocean acidification (Coll‐Lladó, Giebichenstein, Webb, Bridges, & de la serrana, 2018; Comeau et al., 2018; Cornwall et al., 2018; DeCarlo et al., 2017; DeCarlo, Ren et al., 2018; Foster & Clode, 2016; Kamenos et al., 2013; Kamenos, Perna, Gambi, Micheli, & Kroeker, 2016). While these studies have revealed changes in both the mineralogy and disorder of calcified structures under ocean acidification, they have so far conducted Raman spectroscopy analyses of powders or made spot measurements on intact samples. Here, we extend the application of Raman spectroscopy for investigating marine bio‐calcification responses to environmental change with both in vivo and high‐resolution mapping.

Previous Raman studies utilizing spot measurements can resolve mineralogical changes in calcified structures on spatial scales of at least tens to hundreds of μm and on temporal scales of seasons or longer (e.g., Ross et al., 2018). Our in vivo approach enables quantification of mineralogies as well as calcifying fluid Ω*_Ar_* of corals, on a timescale of seconds. This advance can help to resolve the problem of differing temporal and spatial scales among techniques used in coral calcification studies (Holcomb et al., 2014). Although geochemical (i.e., isotopic and elemental) analyses of coral skeletons are effective at deriving the carbonate system of the calcifying fluid (DeCarlo, Holcomb, & McCulloch, 2018; McCulloch, D'Olivo Cordero, Falter, Holcomb, & Trotter, 2017), such studies have been difficult to reconcile with alternative approaches based on inserting micro‐sensors into the coral calcifying fluid (Al‐Horani, Al‐Moghrabi, & De Beer, 2003; Cai et al., 2016; Ries, 2011; Sevilgen et al., 2019), or imaging the calcifying fluid of corals exposed to pH‐sensitive dyes (Comeau et al., 2017; Holcomb et al., 2014; Venn, Tambutte, Holcomb, Allemand, & Tambutte, 2011). Conversely, Raman spectroscopy can now be applied in vivo at temporal and spatial scales comparable to the micro‐sensor studies (Figure 1), as well as to bulk powders for comparison to isotopic measurements (e.g., DeCarlo et al., 2017, DeCarlo, Comeau et al., 2018). Recent development of microfluidic chambers for live‐imaging of coral polyps—“coral‐on‐a‐chip”—(Shapiro, Kramarsky‐Winter, Gavish, Stocker, & Vardi, 2016), could also be readily utilized with in vivo Raman spectroscopy to test acute responses of coral calcifying fluid Ω*_Ar_* and mineralogy to manipulations of seawater chemistry and temperature.

In vivo Raman spectroscopy is especially well‐suited to address the recent debate regarding whether corals precipitate their aragonite skeletons directly from seawater or via transformation of ACC particles (DeCarlo, 2018; DeCarlo, Ren et al., 2018; Von Euw et al., 2017; Mass et al., 2017). Mass et al. (2017) reported ACC in *S. pistillata* based on photoemission electron microscopy, although the ACC was only observed in trace amounts on some outer edges of the skeleton, thus leaving open the possibility that it was an artifact of preparation techniques (including dehydrating the coral, and cutting, grinding, polishing, and embedding the skeleton). Conversely, in vivo Raman spectroscopy does not sacrifice the coral and does not require any preparation of the skeleton, and ACC is easily distinguishable in Raman spectra (Figure 1d). This makes in vivo Raman the ideal technique for determining whether ACC is present in corals. Thus, our finding that ACC was clearly absent from all our in vivo profiles (Figure 2) is key evidence supporting the notion that corals directly precipitate aragonite. While it is important to emphasize that this lack of evidence is not necessarily proof for the complete absence of transient ACC in coral calcification, it indicates that if present, ACC is below the detection limits of our current approach. Importantly, our initial results demonstrate the benefits and ability of Raman spectroscopy to address this question.

High‐resolution ex vivo Raman mapping provides a complementary approach to in vivo profiling. While our in vivo technique provides a snapshot of mineralogy at a single point in time and space, ex vivo mapping reveals the spatial variability of mineralogy and disorder within shells and skeletons (Nehrke, Nouet, & Treude, 2011; Roger et al., 2017; Wall & Nehrke, 2012; Wall, Ragazzola, Foster, Form, & Schmidt, 2015), including banding patterns representing a range of timescales from days to years (Figure 3). Like our in vivo profiling results, high‐resolution ex vivo mapping showed that the corals and fish otolith are composed entirely of aragonite, whereas the foraminifer and coralline alga are entirely calcitic, except for minor aragonite contamination or infilling in the coralline alga. The banding of ν_1_ peak intensity in the fish otolith may be due to differing proportions of organics and aragonite crystals (Jolivet, Bardeau, Fablet, Paulet, & Pontual, 2008, 2013). Conversely, micro‐banding patterns in the aragonitic corals are likely caused by variations in calcifying fluid Ω*_Ar_*, which could also explain the Mg/Ca banding observed here (Figure 3c,d) and described previously (Meibom et al., 2004) because aragonite Mg/Ca increases with Ω*_Ar_* or crystal growth rate (AlKhatib & Eisenhauer, 2017; DeCarlo et al., 2017). Raman bands in the calcitic foraminifer and coralline alga are dominated by the effects of Mg (Figure 3), but it is not yet clear if the Mg banding arises from temporal changes in carbonate chemistry or intrinsic biological rhythms (Fehrenbacher et al., 2017; Spero et al., 2015). Nevertheless, that our Raman‐based visualization of Mg banding in the foraminifer is similar to that from NanoSIMS suggests that Raman spectroscopy can be used as a rapid and cost‐effective technique to map the distribution of Mg in calcitic shells and skeletons.

In summary, Raman spectroscopy adds substantially to the toolbox of techniques available for studying marine bio‐calcification (see Box 1). Among the greatest advantages of Raman spectroscopy is its applicability on small spatial and temporal scales, enabling characterization of near‐instantaneous skeletal growth and micro‐banding patterns produced on various timescales. Although our initial applications provided no evidence for a role of ACC particles in calcification by a range of taxa, Raman spectroscopy can now be readily applied to search for ACC in various developmental stages and under different environmental conditions (Akiva et al., 2018; Foster & Clode, 2016). Organic matrices may also play an important role in calcification for some taxa, and Raman spectroscopy has been used to characterize their distribution in shells and skeletons (DeCarlo, Ren et al., 2018; Von Euw et al., 2017; Jolivet, Bardeau, Fablet, Paulet, & Pontual, 2013; Nehrke et al., 2011). Furthermore, studies of simulated ocean acidification and warming can quantify changes in calcifying fluid Ω*_Ar_* of corals, and mineralogy of coralline algae and foraminifera, without sacrificing experimental replicates (Box 1). For example, in vivo Raman profiling could be conducted repeatedly (e.g., daily) on the same individuals in treatment conditions for weeks to months to assess acclimatization, or on parents and offspring to assess adaptive responses. Since acclimatization and adaptation likely represent critical components of biological responses to global climate change (e.g., Schoepf, Jury, Toonen, & McCulloch, 2017), studying them with Raman spectroscopy will be a valuable research direction.

Box 1 **Potential applications of in vivo Raman spectroscopy for studying marine calcification mechanisms and sensitivities to environmental change [Colour figure can be viewed at http://www.wileyonlinelibrary.com/]**

1 The small spatial and temporal scale of Raman spectroscopy enable its use in experiments ranging from typical control/treatment designs, to acclimatization through life‐history stages, and adaptive responses in multigenerational studies. Since in vivo Raman is non‐destructive, it can be applied to the same individuals over time for some taxa (e.g., corals and coralline algae), but not all (e.g., fish otoliths may only be accessed after sacrificing the organism). Raman spectroscopy provides a variety of information such as mineralogy for all taxa, Mg/Ca ratios for calcitic shells and skeletons, calcifying fluid saturation state of corals, and potentially organic matrix content.

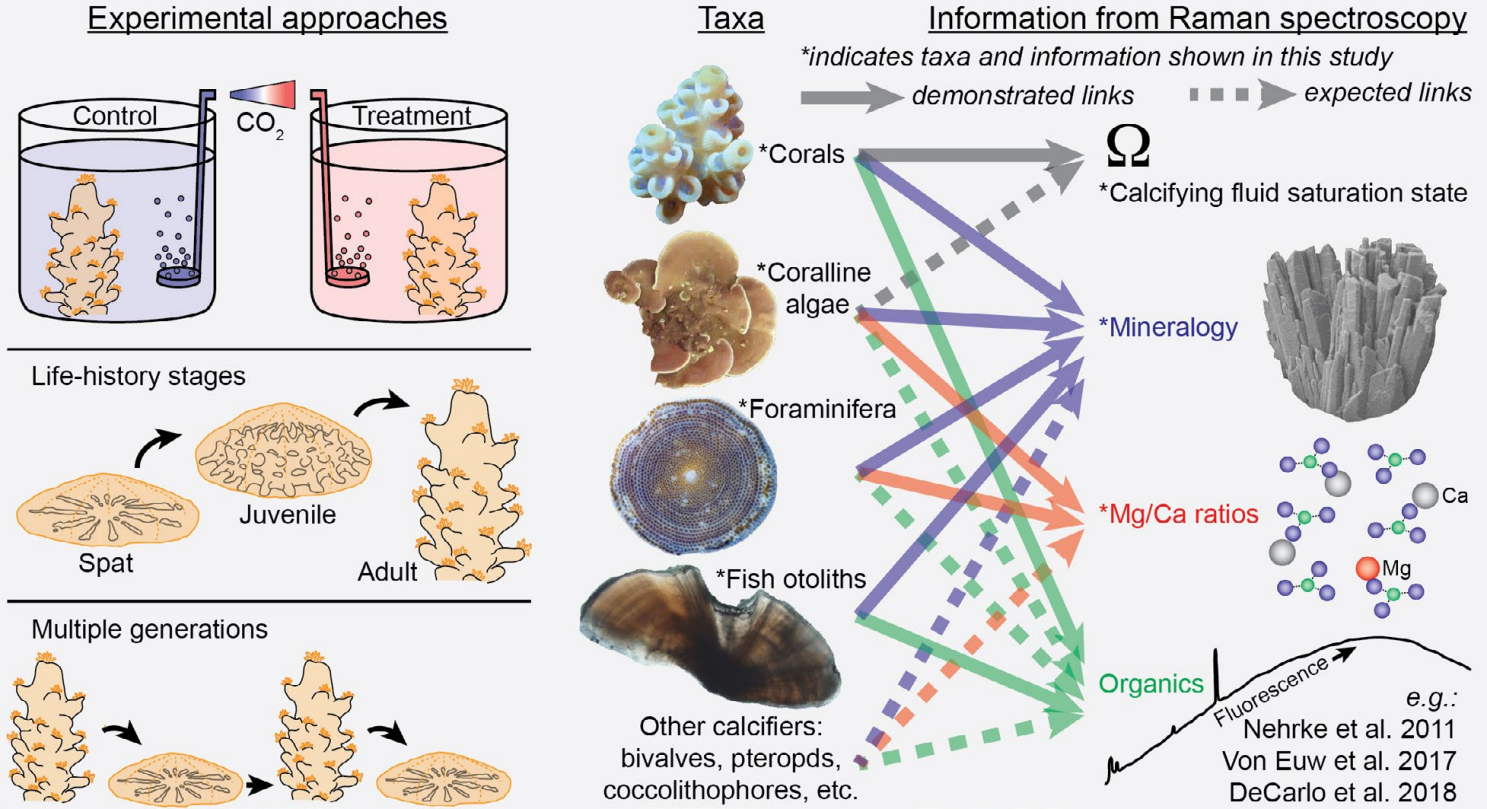

## CONFLICT OF INTEREST

The authors declare no conflicts of interest.
