# Supplementary material for: Investigating marine bio‐calcification mechanisms in a changing ocean with in vivo and high‐resolution ex vivo Raman spectroscopy
Source: Glob Chang Biol. 2019 Feb 20;25(5):1877–88. doi: 10.1111/gcb.14579 (PMC6916197; doi:10.1111/gcb.14579)
Supplement: Supplementary file 1 [file GCB-25-1877-s001.pdf]

Supporting Material for "Investigating marine bio-calcification mechanisms in a changing ocean with *in vivo* and high-resolution *ex vivo* Raman spectroscopy"

NanoSIMS methods

Elemental maps were carried out with a CAMECA NanoSIMS 50L at the Centre for Microscopy, Characterisation and Analysis at the University of Western Australia. The samples were coated with gold to provide conductivity. An O- primary ion beam with a current in the range of 13 pA was used for imaging. Prior to each image, every area was pre-sputtered with the primary beam to a dose of more than 1 x 10^{16} ions/cm^2. The O^- beam was obtained with a Hyperion (H200) RF plasma oxygen ion source. Positive secondary ions ($^{24}Mg^+$ and $^{40}Ca^+$) were collected simultaneously by electron multipliers. Imaging was performed with a raster size of 50 μm^2, a resolution of 512 x 512 pixels and dwell times of 14-15 ms per pixel. Image analysis was carried out using the Fiji plugin OpenMIMS (https://github.com/BWHCNI/OpenMIMS).

Table S1. Integration time, resolution, area, and sampling time of maps shown in Fig. 3

Sample	Integration time (s)	Spatial resolution (μm)	Map area (mm^2)	Mapping time (hr)
S. pistillata 1	2.5	0.25	0.0043	49
S. pistillata 2	1.5	0.25	0.0017	12
D. dianthus	0.5	15	22.5	14
P. fuscus	0.2	1	0.77	43
O. universa	1.2	0.25	0.0036	19
S. durum	0.3	2	1	21

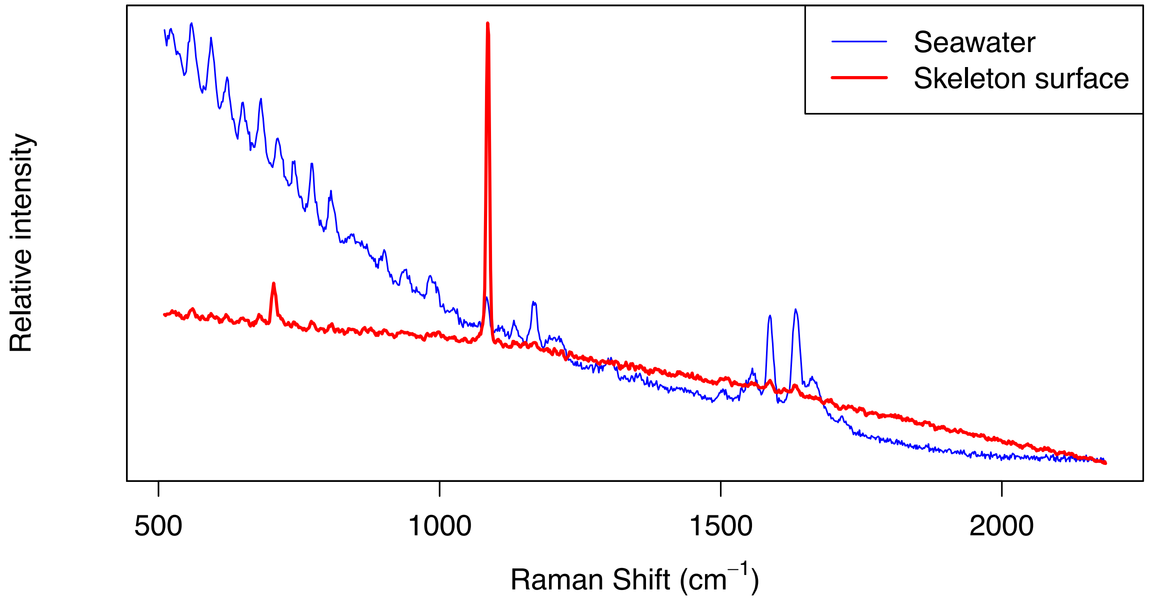

Figure S1. Raman spectra collected with LED lights (Ledzeal Alpha 120W) turned on. The lights caused background fluorescence and several artificial peaks in the Raman spectrum of seawater above the coral polyp (blue line). However, the artefacts of the LED lights were relatively small compared to the aragonite peaks when the focal plane was at the skeletal surface (red line). These spectra were collected in a separate profile from the ones shown in Figs. 1-2, but on the same *A. yongei* 2 specimen.

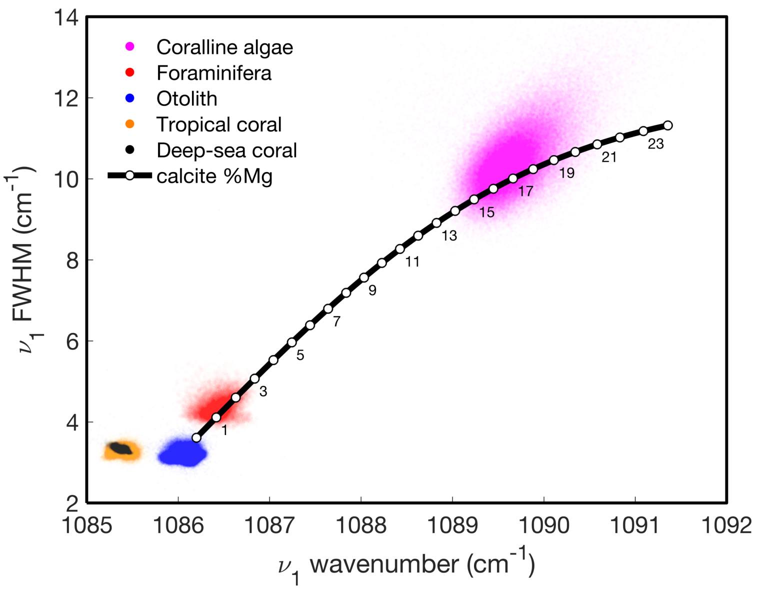

Figure S2. Relationship between ν_1 FWHM and ν_1 wavenumber. Colors show the different organisms for which high-resolution Raman maps were collected (those in Fig. 3). The black line with white points indicates the relationship reported from abiogenic Mg-calcites (Perrin et al., 2016), and the numbers beside the line indicate the expected % Mg. Note that the black line is used to define "residual FWHM". Although the ν_1 wavenumber of the fish otolith is higher than that of the corals, and appears consistent with calcite of very low Mg content, inspection of the ν_4 peak confirmed the sample is entirely aragonitic (see Fig. S3 below).

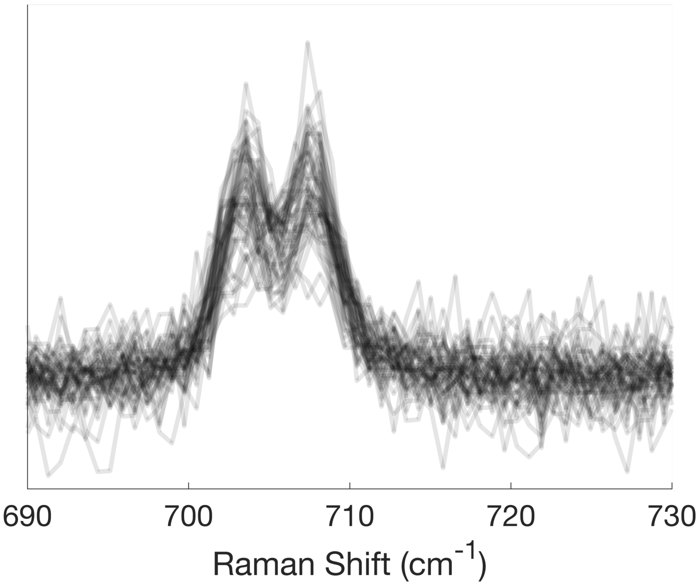

Figure S3. Confirmation that the otolith sample is entirely aragonitic. Inspection of the ν_4 peak shown here for 100 random spectra taken from the map in Fig. 3 shows all the spectra are clearly consistent with the doublet peak of aragonite.
